# Immunotherapy and psychosis: It there a risk?

**DOI:** 10.1192/j.eurpsy.2021.669

**Published:** 2021-08-13

**Authors:** D. Barbosa, M. Mota

**Affiliations:** Psychiatry, Sao Joao Hospital and University Centre, Porto, Portugal

**Keywords:** Immunotherapy, psychosis, Monoclonal antibodies, adverse events

## Abstract

**Introduction:**

Over the past decades, immunotherapy treatments have been a revolution to many chronic diseases with encouraging results in clinical outcomes and quality of life. The use of monoclonal antibodies has yielded a great variability in terms of clinical efficacy and tolerability although it’s believed the incidence of psychotic symptoms is low (0,1-0,4%).

**Objectives:**

To review the effects of monoclonal antibodies on psychosis.

**Methods:**

Review of literature using PubMed database. A total of 16 studies were included.

**Results:**

The targeted molecules by monoclonal antibodies may determine the risk of psychosis. While those who target TNF-alfa seem to have a reduced risk of psychosis (such as Infliximab, Adalimumab, Certolizumab and Golimumab), monoclonal antibodies who modulate lymphocytes may have a greater risk of psychosis namely Natalizumab, Belimumab, Basiliximab and Daclizumab, which seems to correlate to evidence of alterations in lymphocyte subsets in groups of patients with first psychotic episode and schizophrenia. Some seem to have positive correlation with psychosis namely monoclonal antibodies who have a supressing effect on the immune system, especially those who target adaptative immunity and those who are used in autoimmune diseases (vs oncologic conditions). It is unknown if delusions prevail over hallucinations or vice-versa. Despite the paucity of evidence, these findings corroborate the variability regarding the psychiatric effects of immunotherapy.

**Conclusions:**

The available literature reports a low prevalence of psychotic symptoms associated with the use of monoclonal antibodies but it highlights the importance in knowing the immune mechanisms involved in psychotic disorders. Greater research is needed to correctly assess that risk.

